# Polypyrrole coated nonwoven fabric as a piezoresistive sensing material: a multifunctional study on physicochemical, electromechanical, and biological assessment

**DOI:** 10.3389/frobt.2026.1917223

**Published:** 2026-09-10

**Authors:** Hariprasad Ramachandran, Sharmila Nageswaran

**Affiliations:** School of Electronics Engineering, Vellore Institute of Technology, Vellore, Tamil Nadu, India

**Keywords:** antibacterial activity, biocompatibility, piezoresistive pressure sensor, polypropylene nonwoven fabric, polypyrrole

## Abstract

Textile-based pressure sensors for wearable applications require simple fabrication methods, long-term durability, and biocompatibility for skin contact, yet existing sensors often lack comprehensive validation. This study fabricates piezoresistive pressure sensors by depositing polypyrrole (PPy) on polypropylene nonwoven fabric via *in situ* chemical polymerization. This work combines simple fabrication with comprehensive electromechanical and biological validation. Material characterization using XRD, FTIR, Raman, SEM/EDX, and AFM confirmed uniform PPy coatings. The sensors show piezoresistive response across 0.6–6.1 kPa, with quasi-linear voltage increase and exponential resistance decrease under pressure. Voltage response followed a linear fit (sensitivity 0.47 V/kPa, *R* ^2^ = 0.993), while resistance response followed an exponential decay fit (*R* ^2^ = 0.998). Performance testing revealed 1000-cycle durability, one-hour stability (voltage drift −1.64%), temperature-dependent resistance response evaluated over an extended 30 °C–100 °C range for material-level characterization (NTC coefficient −0.941%/°C), and functionality retention over 7 washing cycles. Hysteresis measured 8.68% of full-scale output. Biocompatibility testing (ISO 10993–5) showed cell viability maintained above 70% at all concentrations up to 100 μg/mL, and antibacterial testing (AATCC 147) showed zones of 37–40 mm against *E. coli* and 38–42 mm against *S. aureus*. Overall, these laboratory-scale results indicate that PPy-coated nonwoven fabric is a promising material for durable, biocompatible pressure sensors.

## 1 Introduction

Wearable electronics and smart textiles have gained significant attention in recent years due to their ability to combine the comfort of traditional fabrics with advanced sensing capabilities (Stoppa and Chiolerio, 2014; Chen et al., 2023). Unlike conventional rigid electronic devices, these fabric-based systems can be worn directly on the body, offering continuous monitoring of physiological signals without restricting the user’s movement (Sajovic et al., 2023; Allish et al., 2024). Smart textiles can detect pressure, temperature, and biochemical signals while maintaining the breathability and flexibility expected from everyday clothing (Hassabo et al., 2023; Younes, 2023). By integrating conductive materials into fabrics through coating, weaving, or printing techniques, researchers have developed wearable sensors suitable for healthcare monitoring, sports performance tracking, and human-machine interaction (Allish et al., 2024; Singha et al., 2019).

Among the various fabric-based sensing approaches, piezoresistive pressure sensors have attracted considerable research interest owing to their straightforward transduction mechanism, in which an applied mechanical load induces a measurable change in electrical resistance (Khan et al., 2023; Lipol et al., 2023). In contrast to conventional rigid silicon-based devices, fabric-based piezoresistive sensors offer the advantage of conforming to irregular body contours, preserving wearing comfort, and being seamlessly incorporated into garments without altering their aesthetic or tactile properties (Camlibel and Kandola, 2025; Barati and Nikfarjam, 2025; Razbin et al., 2025). Carbon nanotube networks have been extensively employed as the conductive element in such sensors, primarily because of their exceptional electrical conductivity and their capacity to sustain stable performance under repeated mechanical deformation, including bending and compression (Camlibel and Kandola, 2025). Beyond material selection, scalable fabrication strategies such as Jacquard weaving and spray coating have been successfully demonstrated, facilitating large-area sensor manufacturing with rapid response characteristics and reliable long-term operational stability (Zhao W. et al., 2025; Kim et al., 2022). Continued efforts to enhance sensor sensitivity, broaden the detectable pressure range, and improve environmental stability have driven the exploration of a diverse array of advanced functional materials (Smith et al., 2024; Yin and Sun, 2025). Among these, MXene nanosheets have emerged as particularly promising candidates for piezoresistive applications, attributable to their outstanding electrical conductivity, abundant surface functional groups, and high specific surface area (Li W. et al., 2024). Graphene-coated nonwoven substrates and graphene-impregnated textile composites have demonstrated effective piezoresistive sensing with tunable conductivity and mechanical robustness (Yu et al., 2022; Zhao et al., 2024). Further improvements in sensor performance have been achieved through the incorporation of silver nanoparticles and carbon nanotube-modified nonwoven fabrics, which provide enhanced interfacial contact and improved charge transport pathways (Lu et al., 2019; Jain and Chatterjee, 2024).

More recently, laser-induced graphene patterned directly onto polyimide woven fabrics has enabled the fabrication of multifunctional smart textile garments capable of simultaneous sensing and energy-related functions (Mahmud et al., 2025). Conductive hydrogels incorporating PEDOT:PSS have been investigated as sensing elements for biomedical and wearable applications, offering a compelling combination of high electrical conductivity and mechanical compliance resembling soft biological tissue (Hu et al., 2025). Conducting polymer coatings such as polypyrrole applied onto stretchable textile substrates through low-temperature interfacial polymerization have also been reported as highly sensitive strain sensing platforms (Liu et al., 2025). Complementing these developments, flexible sensors engineered to resolve strain components in multiple directions have been reported, extending the capability of wearable systems for comprehensive human motion monitoring (Huang et al., 2024; Qin et al., 2025). Self-powered multifunctional sensing platforms based on tribovoltaic coatings (Chen et al., 2025) and nanocellulose-integrated flexible electronic architectures (Wang et al., 2026) have further expanded the design space for next-generation wearable devices. Furthermore, functional textile coatings that synergistically incorporate sensing with adaptive moisture and thermal regulation (Zhong et al., 2025), antimicrobial activity (Su et al., 2025), and fully integrated electrochemical sweat biomarker analysis (Zhao Y. C. et al., 2025) are actively being pursued to advance the practicality and clinical relevance of continuous wearable health monitoring systems.

Polypyrrole (PPy) has emerged as one of the most widely studied conductive materials for textile-based pressure sensors, owing to its good electrical conductivity, environmental stability, ease of synthesis, and strong adhesion to fabric surfaces (Chen et al., 2019; Gleissner et al., 2024; Ebadi and Jafari, 2025). It can be deposited onto textiles through *in situ* chemical polymerization or interfacial polymerization, both of which are scalable methods compatible with existing textile processing (Chen et al., 2019; Zahid et al., 2022). The electrical conductivity of PPy-coated substrates is closely governed by polymerization parameters, including oxidant type, monomer concentration, and reaction temperature, as evidenced in PPy-based nanofiber composite systems, where precisely controlled *in situ* polymerization conditions directly determine the uniformity and density of the conductive layer, ultimately influencing the overall sensing performance (Sparavigna, 2017). PPy coatings also maintain their conductivity after repeated mechanical deformation, including bending, stretching, and compression, which is important for reliable wearable sensor performance (Zhou et al., 2022). In addition, PPy shows natural antibacterial activity because its positive charge interacts with the negatively charged bacterial cell membranes, disrupting them. This makes PPy-coated fabrics attractive for sensors that will be in direct contact with the skin for extended periods (Gleissner et al., 2024).

The choice of fabric substrate plays an important role in determining the performance of a piezoresistive sensor. Nonwoven fabrics, which are made by bonding fibres together mechanically or chemically rather than by weaving or knitting, offer several advantages for sensor applications. Their porous three-dimensional fibre structure provides a large surface area for uniform polymer coating, good compressibility for pressure sensing, and dimensional stability under repeated loading (Smith et al., 2024; Yin and Sun, 2025). Nonwoven fabrics are also breathable and allow moisture to pass through, which is important for user comfort during long-term wear and for preventing sweat from degrading the sensor’s electrical performance (Li W. et al., 2024; Yu et al., 2022). From a manufacturing point of view, nonwoven fabrics are cost-effective, widely available in different fibre types and thicknesses, and easy to coat using standard wet-chemical processes, making them suitable for large-scale sensor production (Zhao et al., 2024; Lu et al., 2019; Jain and Chatterjee, 2024). In particular, polypropylene (PP) nonwoven fabric is a strong candidate as a sensor substrate because it is hydrophobic, chemically resistant, and significantly less expensive than cotton or polyester alternatives, making it suitable for low-cost or disposable wearable sensors.

Despite the progress made in this field, several important limitations remain. Many reported sensors have not been thoroughly tested under realistic use conditions. In particular, data on long-term electrical stability under sustained loads, sensor behaviour at different temperatures, performance after washing, and quantitative hysteresis analysis are rarely provided (Zhou et al., 2018). Although PPy-coated sensors have been fabricated on cotton and polyester substrates (Chen et al., 2019; Gleissner et al., 2024), studies that combine thorough mechanical durability testing, long-term stability evaluation, and formal biocompatibility assessment are rarely reported in the literature (Cao, 2024). Moreover, many fabrication methods involve multiple complex steps or specialized equipment that are difficult to scale up for commercial production. While some studies have explored combining sensing and antibacterial functions in textiles (Li Y. et al., 2024; Aizamddin et al., 2022; Aizamddin et al., 2024), comprehensive validation of both antibacterial activity and cytotoxicity according to recognized international standards such as ISO 10993–5 and AATCC 147 is rarely reported together in a single study.

This work addresses these gaps by systematically characterising PPy-coated polypropylene nonwoven fabric pressure sensors. The key novelties of this study are the use of a polypropylene nonwoven substrate, which offers better moisture resistance and lower cost compared to commonly used cotton or polyester substrates; a straightforward, single-step *in situ* polymerization method carried out under static conditions, which produces uniform PPy coatings without the need for complex equipment or multiple processing steps; and the combination of comprehensive electromechanical characterization with standardized biocompatibility and antibacterial validation. To ensure thorough characterization, sensors were tested over 1000 loading–unloading cycles, monitored for 1 hour under a constant load, evaluated across a temperature range of 30 °C–100 °C, assessed after seven washing cycles, and analysed for hysteresis using a full-scale output method. Biocompatibility was validated through ISO 10993–5 cytotoxicity testing and AATCC 147 antibacterial assessment against both Gram-positive and Gram-negative bacteria. The sensors demonstrated a working pressure range of 0.6–6.1 kPa, corresponding to pressures relevant to applications such as human touch, finger motion, and joint bending. The overall aim of this work is to develop a practical and scalable approach for producing durable, biocompatible, and multifunctional textile pressure sensors for wearable applications.

## 2 Materials and methods

### 2.1 Material

The pyrrole monomer (C4H5N, 99% purity), sodium hydroxide (99% purity) was purchased from the Sisco Research Laboratories, India, ferric chloride (FeCl3, 99% purity) was purchased from the Molychem, India, and para-toluenesulphonic acid (pTSA, 99% purity) were purchased from the Nice Chemicals, India. A carbon-impregnated polyethylene conductive film (thickness: 0.09 mm) was purchased from Thingbits Electronics, India. The substrate material consisted of polypropylene (PP) spunbond non-woven fabric with the following specifications: surface density of 24 g/m^2^, thickness of 0.1 mm, fibre diameter range of 10–12 μm, and an estimated porosity of ∼73.5%, calculated from the fabric’s areal density and thickness relative to the bulk density of polypropylene (0.905 g/cm^3^), consistent with its highly porous, breathable structure. The non-woven fabric was sourced from Sivasamy Silks, India. The chemicals used in this research study were analytical grade and used without any further purification. The entire experiment used deionized water.

### 2.2 Methods

#### 2.2.1 Synthesis of PPy

**FIGURE 1:**
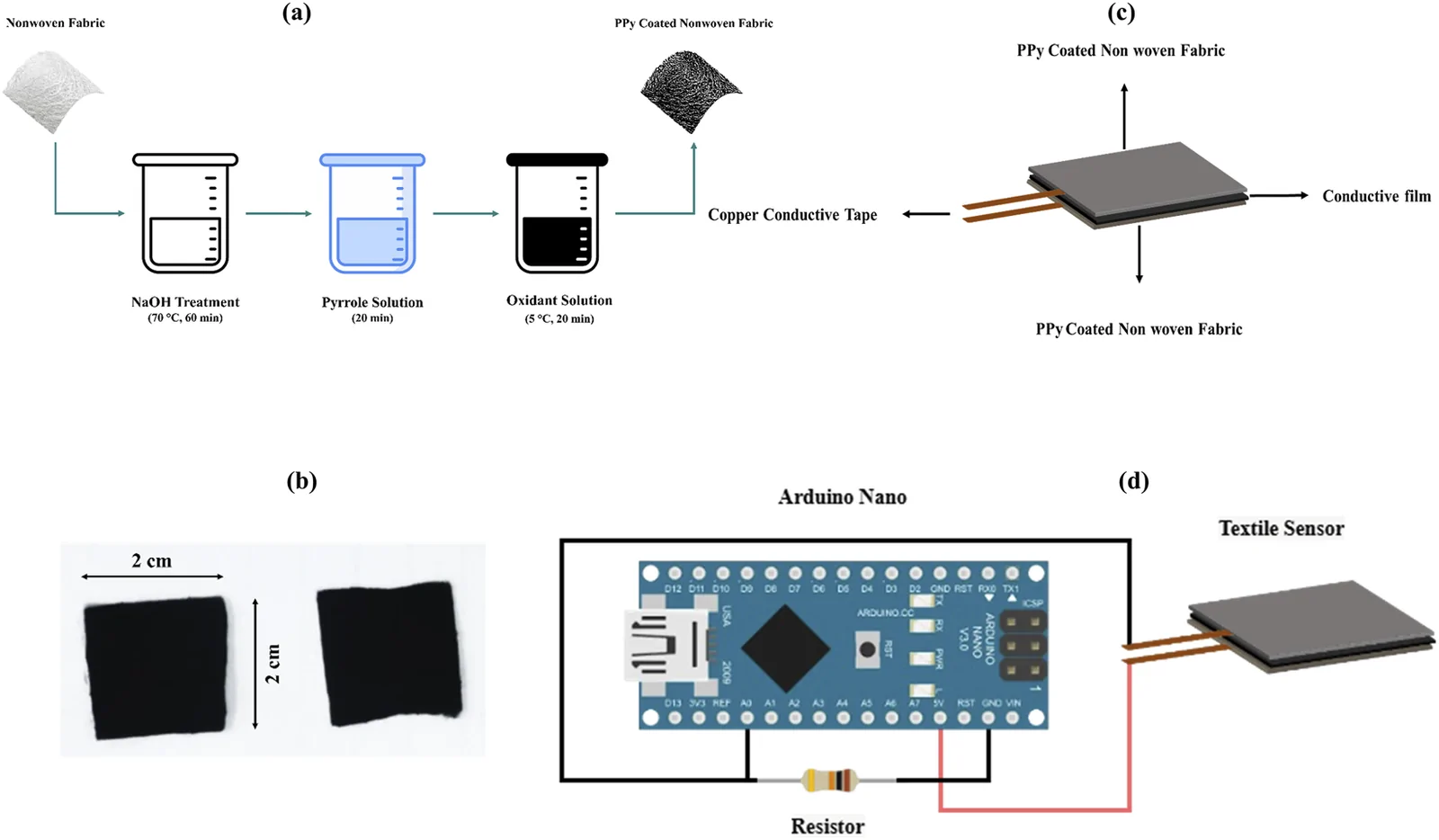
**(a)** Schematic illustration of PPy coating process on polypropylene nonwoven fabric through *in situ* chemical polymerization: (i) NaOH pretreatment at 70 °C, (ii) pyrrole monomer absorption at room temperature, (iii) oxidative polymerization with FeCl_3_/pTSA at 5 °C, and (iv) washing and drying at 60 °C. **(b)** Photograph showing a PPy-coated non-woven fabric sample prepared by *in situ* chemical polymerization. **(c)** Sandwich-type sensor structure comprising PPy-coated nonwoven fabric as top and bottom electrodes with carbon-impregnated polyethylene film as intermediate conductive layer, with copper tape electrical contacts. **(d)** Schematic diagram of voltage divider circuit configuration for resistance measurement showing 5V power supply, textile sensor, 10 kΩ reference resistor, and Arduino Nano analog input (10-bit ADC) for data acquisition. The junction voltage between the sensor and reference resistor is measured to calculate sensor resistance.

Initially, the non-woven fabric (2 cm × 2 cm) was cleaned to eliminate surface contaminants. A 37.5 mM sodium hydroxide solution (500 mL) was prepared and heated to 70 °C. The fabric was immersed in the solution for 60 min, then it was removed and gently cleaned with deionized water. The cleaned fabric was subsequently immersed in a 0.5 M pyrrole monomer solution (100 mL, prepared in deionized water) for 20 min at the ambient temperature to promote monomer diffusion into the fabric in order to create PPy. An oxidant solution was formed separately using 0.02 M of p-toluenesulfonic acid (pTSA), and 1 M of ferric chloride (FeCl_3)_ in deionized water (50 mL) as per the literature (Maity and Chatterjee, 2015; Maity and Chatterjee, 2014). After monomer absorption, the fabric was transferred to the pre-cooled oxidant solution and maintained at 5 °C for 20 min under static conditions to initiate and complete the *in situ* oxidative polymerization of pyrrole on the fiber surfaces. The PPy-coated fabric was then retrieved, and carefully washed with deionized water to remove remaining oxidant and by-products, and dried in an oven at 60 °C for 2 h (Figure 1a). Mass measurements before (0.0120 g) and after (0.0417 g) coating indicated a PPy mass of 29.7 mg. The resulting conductive fabric (Figure 1b) exhibited uniform black coloration characteristic of PPy and was stored in a clean, dry environment for subsequent characterization and sensor fabrication.

#### 2.2.2 Characterization techniques

The PPy-coated non-woven fabric was characterized using several techniques. X-ray diffraction (XRD) analysis (Bruker D8 Advance with Cu Kα radiation, λ = 1.54056 Å) was performed over a 2θ range of 10°–90° with a step size of 0.02° and a counting time of 1 s/step, to determine the sample’s crystalline nature and phase purity. UV-visible spectroscopy (Specord, 210 plus) was recorded over a wavelength range of 200–800 nm to determine the optical properties. Fourier transform infrared (FTIR) spectroscopy (JASCO, 4600) was recorded over a spectral range of 500–4000 cm^-1^ at a resolution of 8 cm^-1^, averaging 64 scans, to provide insights into surface functional groups. Raman spectroscopy (Horiba France, XploRA PLUS) was performed using a 532 nm laser source over a spectral range of 400–2000 cm^-1^ to study the structural properties of the PPy nanoparticles. Scanning electron microscopy coupled with energy-dispersive X-ray (SEM/EDX) spectroscopy (EVO 18 Research) was operated at an accelerating voltage of 10 kV and a working distance of 11.5–12.5 mm to analyse the morphological features and elemental composition. An Atomic Force Microscope (AFM) (Nanosurf) operating in static force (contact) mode with a ContAl-G cantilever was used to analyse the surface topography and morphology. Additionally, a semiconductor parameter analyser (SCS 4200 Keithley) was used to determine the sheet resistance via a linear, equally spaced four-point probe configuration, with current sourced through the outer probes and voltage measured across the inner probes.

#### 2.2.3 Sensor fabrication

The piezoresistive sensor was constructed in a sandwich-type layered structure (Figure 1c) comprising PPy-coated non-woven fabric as both the bottom and top conductive electrodes, with a carbon-impregnated film serving as a supportive interlayer. The PPy-coated fabric, prepared via *in situ* chemical polymerization as described in Section 2.2.1, functioned as flexible conductive electrodes while providing structural support. All layers were aligned and cut to uniform dimensions of 2 cm × 2 cm, providing a sensing area of 4 cm^2^.

Copper conductive adhesive tape was affixed along one edge of both the top and bottom PPy-coated fabric layers to serve as electrical contacts. Electrical connections were established by attaching insulated test clips to the copper tape, ensuring stable contact and consistent signal transmission during measurements. The assembled sensor was mounted on a flat, rigid platform for testing (Wang et al., 2022; Xie et al., 2025).

#### 2.2.4 Electrical measurement

Electrical resistance was measured using a voltage divider configuration (Figure 1d). One sensor lead was connected to the 5V supply, while the other sensor lead connected to one terminal of a 10 kΩ reference resistor, with the resistor’s opposite terminal grounded. The junction voltage between the sensor and resistor was measured using the Arduino Nano’s analog input (10-bit ADC, 0–1023 corresponding to 0–5V). Resistance values were recorded after the displayed reading stabilized visually, typically within 5–10 s following load application.

#### 2.2.5 Mechanical testing

Mechanical loading was applied using calibrated weights of 50, 100, 150, 200, 250, 300, 350, 400, 450, and 500 g, added in ascending order. Each weight was placed carefully onto the sensor surface over ∼2–3 s. The equivalent pressures were calculated based on the applied force and the sensing area. At each loading stage, the resistance across the sensor was recorded using the Arduino Nano connected to a computer for data acquisition, following the stabilization protocol described in Section 2.2.4.

The loading and unloading cycles were performed manually, with weights removed one by one in reverse order (from 500 g down to 50 g) using the same gentle removal method as during loading. Hysteresis behaviour was evaluated by comparing resistance values during loading and unloading sequences (Wei et al., 2024; Trung and Lee, 2016). Dynamic characteristics, including response and recovery times, were assessed by rapidly applying and removing a 100 g load while continuously monitoring resistance changes.

#### 2.2.6 Washability testing

The durability of the PPy coating under repeated washing was evaluated in accordance with the AATCC 135 standard test method for dimensional changes of fabrics after domestic laundering.

#### 2.2.7 Antibacterial testing

The antimicrobial performance of the PPy-coated fabric was evaluated using the AATCC 147 Parallel Streak Method against *Escherichia coli* (Gram-negative) and *Staphylococcus aureus* (Gram-positive). Bacterial cultures were grown to a concentration of 1.5 × 10^8^ CFU/mL and streaked in five parallel lines across nutrient agar plates. Test specimens measuring 1 cm × 1 cm, including two identical PPy-coated fabric replicates (S1 and S2, prepared under the same polymerization conditions) and an uncoated control fabric, were UV-sterilized for 30 min, then positioned perpendicular to the bacterial streaks with gentle pressure to ensure contact. The inoculated plates were incubated at 37 °C for 24 h, after which zones of inhibition perpendicular to the streaks were measured.

#### 2.2.8 Cytotoxicity testing

The biocompatibility of the PPy-coated fabric was assessed in accordance with ISO 10993–5, using the MTT assay with L929 mouse fibroblast cells. Cells obtained from the National Centre for Cell Science (Pune, India) were maintained in Dulbecco’s Modified Eagle Medium supplemented with 10% fetal bovine serum and 1% penicillin-streptomycin under standard culture conditions (37 °C, 5% CO_2_, humidified atmosphere). For the cytotoxicity test, L929 cells were seeded at a density of 1 × 10^4^ cells per well in 96-well plates and allowed to attach for 24 h. Sample extracts were prepared by immersing 10 mg of PPy-coated fabric in 1 mL of culture medium at 37 °C for 24 h, followed by sterile filtration. The extract was then serially diluted to obtain test concentrations of 0, 20, 40, 60, 80, and 100 μg/mL, where 0 μg/mL served as the negative control. Each concentration was tested in triplicate (n = 3). Following 24-h exposure to the extracts, cell viability was quantified using the MTT assay. MTT solution (10 μL, 5 mg/mL in PBS) was added to each well and incubated for 4 h to allow formazan crystal formation. The crystals were subsequently dissolved in 100 μL dimethyl sulfoxide, and absorbance was measured spectrophotometrically at 570 nm. Cell viability was calculated as the percentage of sample absorbance relative to control absorbance.

## 3 Results and discussion

### 3.1 XRD analysis

**FIGURE 2:**
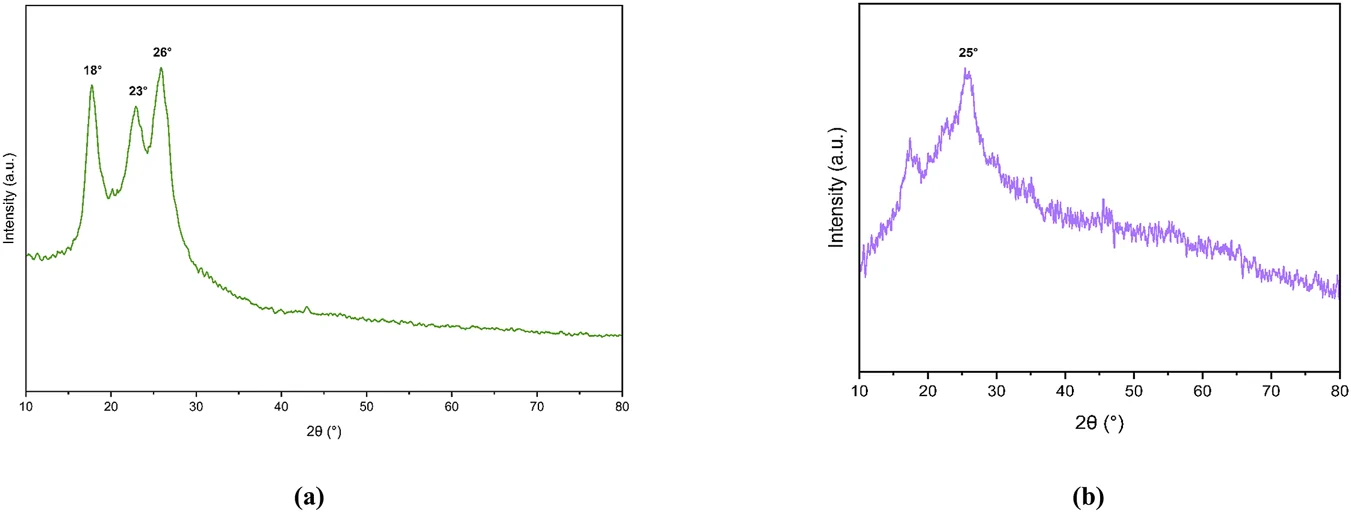
XRD patterns of pristine polypropylene nonwoven fabric **(a)** and PPy-coated fabric **(b)**. Pristine fabric shows characteristic crystalline peaks at 2θ = 18°, 23°, and 26° corresponding to (110), (040), and (130) planes of α-polypropylene. PPy-coated sample exhibits a broad peak centered at 2θ ≈ 25° characteristic of an amorphous PPy structure, with attenuated polypropylene substrate peaks indicating successful PPy coating.

XRD analysis was conducted to verify PPy formation on the non-woven fabric and evaluate its structural properties. Figure 2 displays the diffraction patterns for both the uncoated polypropylene fabric and the PPy-coated sample. The uncoated fabric displays sharp diffraction peaks at 2θ values of 18°, 23°, and 26°, which are typical of semi-crystalline polypropylene. These peaks represent the crystallographic reflections from the (110), (040), and (130) planes of α-polypropylene, confirming the crystalline nature of the substrate.

After PPy deposition, the diffraction pattern exhibits a prominent broad peak centered at 2θ ≈ 25°. This broadened feature is typical of chemically synthesized PPy and relates to the (002) plane, indicating limited ordering of the conjugated polymer chains along the c-axis (Negarestani et al., 2024; Yildiz, 2015). The diffuse nature of this peak demonstrates the largely amorphous character of PPy formed through *in situ* polymerization on textile fibres. The appearance of this peak around 25° suggests some degree of π-stacking alignment among PPy chains, though without extensive crystalline ordering, which is expected for PPy prepared by oxidative polymerization. An important observation is the reduction in intensity of the polypropylene substrate peaks (18°, 23°, 26°) in the coated sample. This attenuation indicates that the PPy layer significantly contributes to the overall diffraction signal, effectively diminishing the substrate’s crystalline signature (Negarestani et al., 2024; Yildiz, 2015). This finding confirms adequate PPy coverage on the fabric surface. The diffraction behaviour aligns with reported patterns for PPy synthesized with ferric chloride, which characteristically show broad features between 20° and 30°, with maximum intensity near 25°–26° due to inter-chain π–π interactions (Yildiz, 2015; Das et al., 2024). The presence of this characteristic broad peak confirms successful PPy coating on the non-woven substrate.

### 3.2 FTIR analysis

**FIGURE 3:**
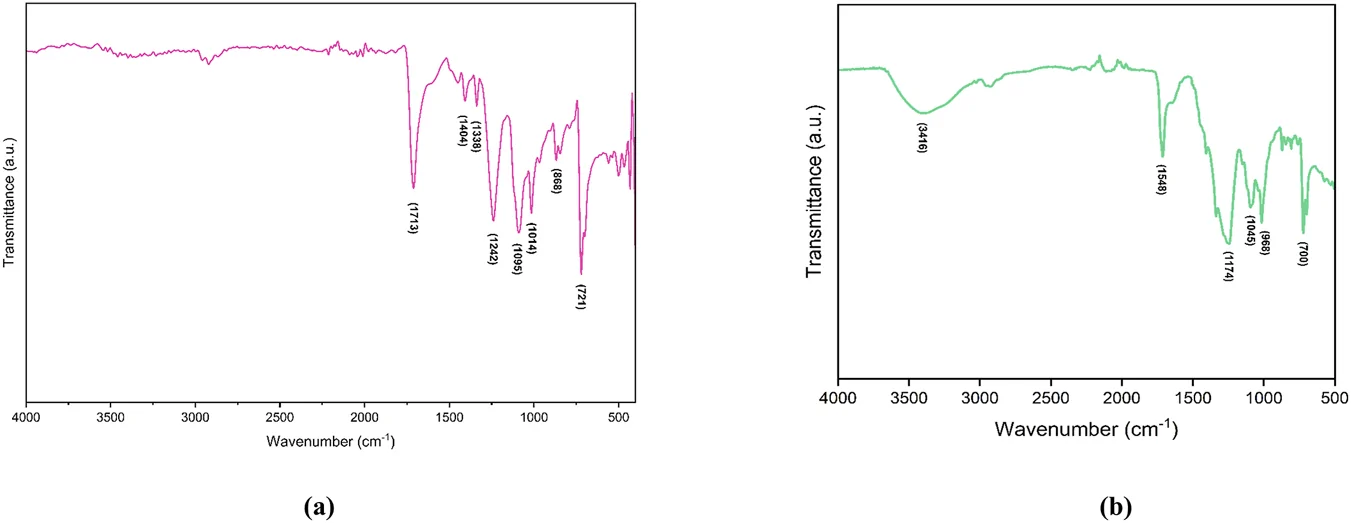
FTIR spectra of pristine polypropylene nonwoven fabric **(a)** and PPy-coated fabric **(b)**. Pristine fabric shows characteristic polypropylene peaks at 1404, 1338, 1095, 868, and 721 cm^-1^. PPy-coated sample displays characteristic absorption bands at 3416 cm^-1^ (N-H stretching), 1548 cm^-1^ (C=C stretching), 1174 and 1045 cm^-1^ (C-H bending), 968 cm^-1^ (C-H out-of-plane deformation), and 700 cm^-1^ (ring deformation), confirming successful PPy deposition.

FTIR spectroscopy was performed to confirm PPy formation and identify functional groups present on both the pristine and PPy-coated non-woven fabrics. Figure 3 presents the comparative FTIR spectra of the uncoated polypropylene fabric and the PPy-coated sample. The pristine polypropylene fabric exhibits characteristic absorption bands at 1404 cm^-1^, 1338 cm^-1^, 1095 cm^-1^, 868 cm^-1^, and 721 cm^-1^, corresponding to CH_3_ bending, CH_3_ symmetric deformation, C-C stretching, and CH_2_ rocking vibrations typical of the polypropylene polymer backbone. These peaks confirm the identity of the substrate material.

Following PPy coating, the FTIR spectrum shows distinct new absorption bands characteristic of polypyrrole, while the polypropylene substrate peaks are significantly diminished. A broad absorption band appears at 3416 cm^-1^, attributed to N–H stretching vibrations of the pyrrole ring structure, which is distinctive of PPy (Sood et al., 2024; Gholse, 2014). The peak at 1548 cm^-1^ corresponds to C=C stretching vibrations associated with the conjugated pyrrole ring system, confirming the presence of the polymer chain structure. The bands at 1174 cm^-1^ and 1045 cm^-1^ are assigned to in-plane C–H bending vibrations characteristic of the aromatic pyrrole ring. The absorption at 968 cm^-1^ is associated with C–H out-of-plane deformation modes, while the peak observed at 700 cm^-1^ corresponds to ring deformation vibrations of the pyrrole structure.

These characteristic absorption bands correlate well with FTIR spectra reported for chemically synthesized PPy, verifying successful coating on the non-woven fabric substrate. The appearance of broad bands and slight peak shifts compared to pure PPy result from interactions between the PPy chains and dopant ions (pTSA) incorporated during synthesis. The dominance of PPy-specific peaks in the coated sample, coupled with the suppression of substrate signals, indicates substantial PPy deposition on the fabric surface (Sood et al., 2024; Kato et al., 1991). The FTIR data, in conjunction with XRD results, confirm the formation of a conductive PPy layer on the non-woven substrate.

### 3.3 Raman spectroscopy analysis

**FIGURE 4:**
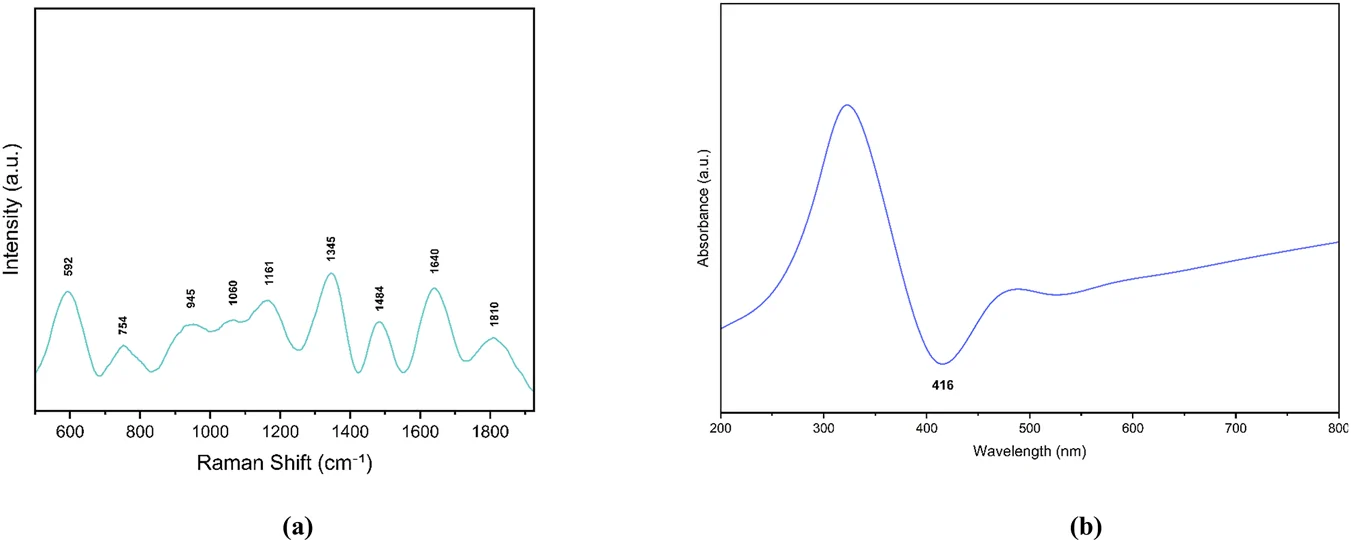
**(a)** Raman spectrum of PPy-coated nonwoven fabric showing characteristic PPy bands at 592 cm^-1^ (ring deformation), 1161 cm^-1^ (C-H bending), 1345 cm^-1^ (C-C and C-N stretching with polaron structures), 1484 cm^-1^ (C=C and C-N stretching), and 1640 cm^-1^ (C=C backbone stretching). Polypropylene substrate signals appear at 754, 945, and 1060 cm^-1^, confirming successful PPy coating on fabric. **(b)** UV-Vis absorption spectrum of PPy-coated polypropylene nonwoven fabric showing characteristic π-π* transition peak at ∼300 nm and broad continuous absorption across the 400–800 nm region. The extended visible-NIR absorption indicates polaron and bipolaron charge carriers characteristic of doped conductive PPy, with an absorption minimum at 416 nm marking the transition region.

Raman spectroscopy was conducted to confirm PPy formation and assess its structural properties. The Raman spectrum of the PPy-coated non-woven fabric is displayed in Figure 4a. The spectrum reveals several distinct bands originating from both the PPy coating and the polypropylene substrate. A band at 592 cm^-1^ corresponds to ring deformation vibrations characteristic of the PPy structure (Nguyen Thi Le, 2004; Vigmond et al., 1995). Bands appearing at 754 cm^-1^, 945 cm^-1^, and 1060 cm^-1^ arise from the polypropylene substrate and are attributed to CH_2_ rocking, CH_3_ rocking, and C-C stretching vibrations, respectively. The signal at 1161 cm^-1^ relates to C-H in-plane bending vibrations of PPy (Vigmond et al., 1995; Trchová and Stejskal, 2018). A prominent band at 1345 cm^-1^ indicates combined C-C ring stretching and C-N stretching vibrations within the PPy backbone, suggesting the presence of polaron structures formed during chemical oxidation (Trchová and Stejskal, 2018; Mikat et al., 2002; Irfan et al., 2024). The band at 1484 cm^-1^ reflects C=C and C-N stretching vibrations in the polymer chain. A strong signal at 1640 cm^-1^ corresponds to C=C stretching vibrations along the conjugated backbone, verifying the presence of oxidized PPy (Nguyen Thi Le, 2004; Liu and Hwang, 2000). An additional band at 1810 cm^-1^ is attributed to an overtone or combination mode within the polymer structure.

The simultaneous observation of PPy-specific bands and substrate signals is expected for polymer-coated textile materials, as the Raman measurement detects contributions from both layers. The characteristic PPy signals, especially those at 1345 cm^-1^ and 1640 cm^-1^, verify successful polymer deposition on the fabric substrate. These spectral observations confirm the formation of conductive PPy on the polypropylene non-woven fabric, demonstrating the material’s potential for sensor applications.

### 3.4 UV-Vis spectroscopy analysis

UV-visible spectroscopy was conducted to investigate the optical properties and electronic structure of the PPy-coated non-woven fabric. Figure 4b displays the UV-Vis absorption spectrum of the coated sample. The spectrum exhibits a strong absorption peak in the UV region around 300 nm, attributed to π-π* electronic transitions within the conjugated PPy backbone (Pawar et al., 2025; Chougule et al., 2011). This characteristic absorption confirms the presence of conjugated π-electron systems along the polymer chain. The spectrum shows a distinct absorption minimum at 416 nm, representing the transition region between the UV absorption band and the extended visible-NIR absorption. Broad, continuous absorption is observed across the visible and near-infrared regions extending from 400 to 800 nm. This extended absorption is characteristic of doped, conductive PPy and arises from polaron and bipolaron charge carriers formed during the oxidative polymerization process with FeCl_3_ and pTSA (Pawar et al., 2025; Chougule et al., 2011). The presence of these charge carriers indicates successful *in situ* polymerization and confirms the doped state of PPy on the fabric substrate. The gradually increasing absorption tail in the NIR region is consistent with free carrier absorption associated with the conductive state of the polymer coating.

The observed spectrum represents the combined optical response of both the PPy coating and the underlying polypropylene substrate. The dominant PPy-related features, particularly the UV absorption and the broad visible-NIR tail, confirm substantial polymer deposition on the fabric. These spectral characteristics align with previously reported UV-Vis behaviour of chemically synthesized PPy on textile substrates, validating successful coating and doping of PPy on the non-woven fabric and supporting the material’s suitability for electronic and sensing applications.

### 3.5 SEM and EDX analysis

**FIGURE 5:**
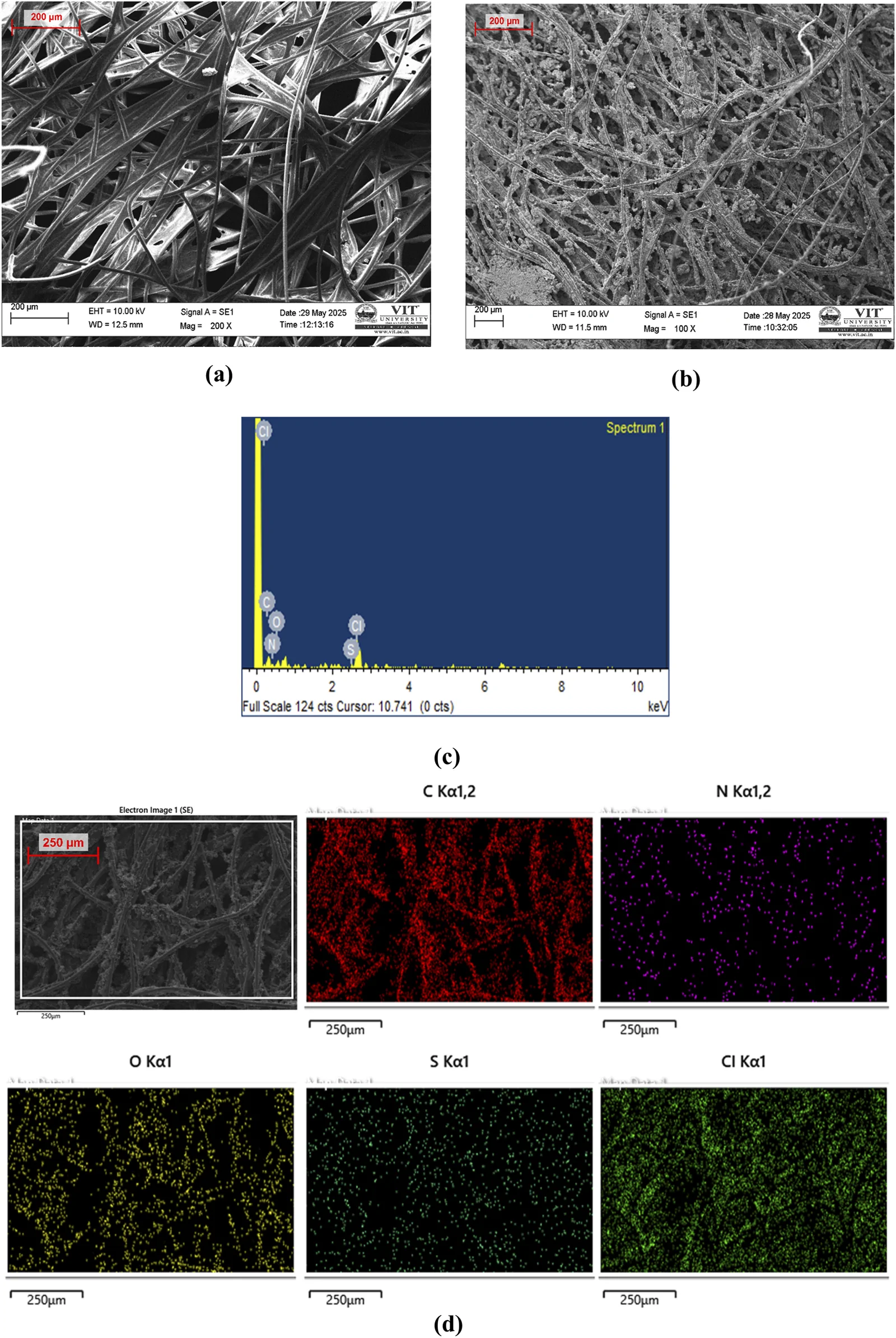
SEM images and EDX analysis of **(a)** pristine polypropylene nonwoven fabric with smooth fibre surfaces and porous three-dimensional structure (×200 magnification, scale bar: 200 μm), **(b)** PPy-coated fabric exhibiting granular morphology with uniform PPy particle coverage on fibre surfaces (×100 magnification, scale bar: 200 μm), **(c)** EDX spectrum of the PPy-coated fabric confirming the presence of carbon (C), nitrogen (N), oxygen (O), sulfur (S), and chlorine (Cl), and **(d)** EDX elemental distribution maps showing the spatial distribution of C, N, O, S, and Cl across the coated fabric surface (scale bar: 250 μm), confirming consistent PPy coating deposition throughout the fibre network.

The surface morphology and elemental composition of the pristine and PPy-coated non-woven fabrics were investigated using scanning electron microscopy (SEM) and energy-dispersive X-ray spectroscopy (EDX). Figure 5 presents the SEM micrographs, EDX spectrum, and EDX elemental distribution maps. The SEM image of the pristine polypropylene fabric (Figure 5a) reveals uniform, smooth cylindrical fibres with clean surfaces devoid of particulate matter or surface deposits. The fibres exhibit a relatively smooth morphology typical of spunbond polypropylene non-woven materials. Following *in situ* polymerization, the PPy-coated fabric (Figure 5b) displays a distinct change in surface texture. The fibre surfaces are covered with a granular coating of PPy, forming nodular structures that envelope the fibres while preserving the underlying fibrous architecture. This granular morphology is characteristic of PPy synthesized via chemical oxidative polymerization on textile substrates, where nucleation and growth processes result in the formation of discrete PPy particles on the fibre surfaces (Saini and Choudhary, 2013). The preservation of the fabric’s porous structure is evident from the SEM images, indicating that the coating process does not visibly obstruct the underlying pore network.

EDX spectral analysis (Figure 5c) confirms the successful deposition of PPy and associated chemical species on the coated fabric. The spectrum displays characteristic elemental peaks corresponding to carbon (C), nitrogen (N), oxygen (O), sulfur (S), and chlorine (Cl). Quantitative analysis (Table 1) reveals that carbon constitutes the major component (68.94 wt%), consistent with the polypropylene substrate and the PPy backbone. The detection of nitrogen (6.38 wt%) is particularly significant, as it directly confirms the incorporation of pyrrole units in the coating, given that nitrogen is entirely absent in the pristine polypropylene substrate (Pattananuwat et al., 2021; Lee, 2024). Sulfur (0.17 wt%) originates from the p-toluenesulfonic acid dopant incorporated during polymerization. The presence of chlorine (17.36 wt%) is attributed to residual iron chloride from the FeCl_3_ oxidant used in the synthesis. Oxygen (7.15 wt%) may arise from atmospheric oxidation of the PPy surface or trace moisture absorption (Lee, 2024; Cucchi et al., 2009).

**TABLE 1:** Elemental composition from EDX analysis.

Element	Weight %	Atomic %	Assignment
C	68.94	80.42	PP substrate + PPy backbone
N	6.38	6.38	PPy (pyrrole units)
O	7.15	6.26	Surface oxidation
S	0.17	0.07	pTSA dopant
Cl	17.36	6.86	Residual FeCl_3_

To further verify the spatial distribution of elements across the coated fabric surface, EDX elemental mapping was performed and is presented in Figure 5d. The maps show carbon (C Kα1,2) distributed throughout the fibre network, matching the expected contribution of the polypropylene substrate and PPy backbone. Nitrogen (N Kα1,2) is present along the fibre surfaces, supporting that PPy coating extends across the fabric rather than being localized to specific regions. Oxygen (O Kα1), sulfur (S Kα1), and chlorine (Cl Kα1) show a similar dispersed pattern across the mapped area. The spatial co-distribution of nitrogen, sulfur, and chlorine with the fibre structure indicates that the PPy layer is continuous and well-adhered to the fabric substrate. Together, the SEM and EDX results confirm successful PPy coating of the non-woven fabric substrate, reflecting the granular morphology observed in SEM images and the elemental signatures and spatial distribution captured by EDX. This surface coverage supports the material’s suitability for flexible piezoresistive sensing applications.

### 3.6 AFM analysis

**FIGURE 6:**
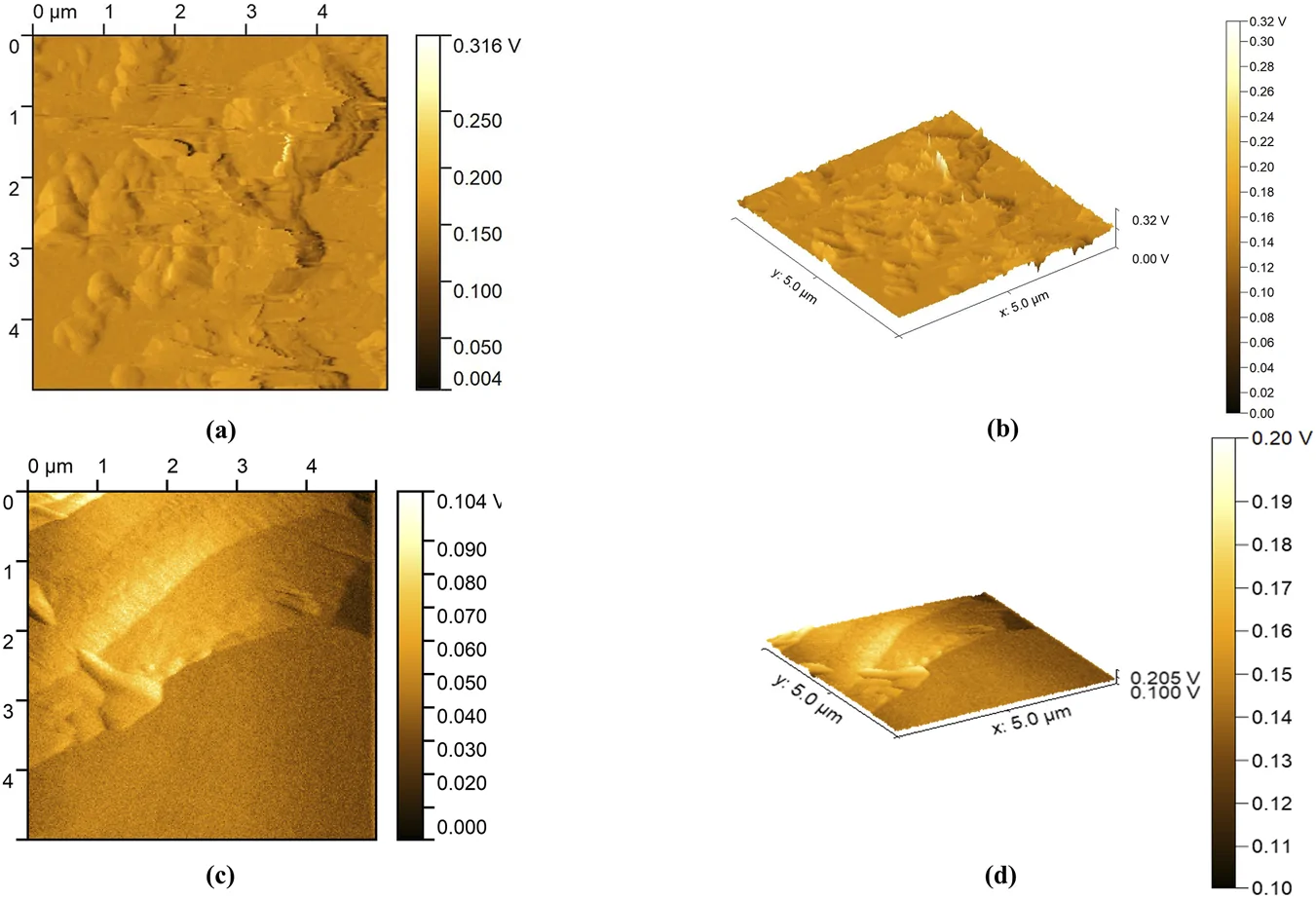
AFM topography comparison of **(a,b)** PPy-coated and **(c,d)** pristine polypropylene fabric, shown as 2D height maps **(a,c)** and 3D views **(b,d)** (scan: 5.0 μm × 5.0 μm). The pristine fabric shows a comparatively smooth surface (Ra = 97.85 nm, Rq = 110.1 nm), while the PPy-coated fabric exhibits pronounced nanoscale roughening (Ra = 293 nm, Rq = 380 nm), consistent with the granular, nodular PPy morphology enhancing effective inter-fibre contact area for piezoresistive sensing.

Atomic force microscopy (AFM) was employed to investigate the nanoscale surface morphology and roughness of the PPy-coated non-woven fabric. Topographic measurements were performed in static force mode using a ContAl-G cantilever over a scan area of 5.0 μm × 5.0 μm. Figure 6a-d) presents the resulting two- and three-dimensional AFM topography images of the PPy-coated fabric surface, revealing a nodular morphology characteristic of chemically synthesized PPy coatings (Pelto et al., 2013; Berendjchi et al., 2016). Quantitative roughness analysis yielded an arithmetic average roughness (Ra) of 293 nm and a root mean square roughness (Rq) of 380 nm, with a maximum peak height (Rmax) of 1753 nm and a maximum valley depth (Rmin) of −1332 nm, indicating substantial surface variation introduced by the coating. For comparison, AFM analysis of the pristine, uncoated polypropylene fabric yielded a markedly lower Ra of 97.85 nm and Rq of 110.1 nm, with a maximum peak-to-valley height (Rt) of 424.6 nm. This disparity confirms that the nanoscale roughness observed after polymerization arises from the nucleation and aggregation of PPy particles on the fibre surfaces during *in situ* oxidative polymerization, rather than from the underlying fabric structure.

This quantified increase in surface roughness is expected to enhance the effective contact area of the coated fabric, which is advantageous for piezoresistive sensing applications, as it promotes improved inter-fibre contact under applied pressure. AFM measurements on highly porous, three-dimensional non-woven fabrics present challenges due to the irregular surface topology; nonetheless, the scanned region offers a representative view of the PPy coating morphology on individual fibre surfaces (Berendjchi et al., 2016). The nodular PPy morphology observed within this scanned area is consistent across both the 2D and 3D images. The AFM findings corroborate the SEM observations and align with literature reports on PPy synthesis on flexible substrates via chemical oxidative polymerization.

### 3.7 Four-probe electrical characterization

The electrical properties of the coated non-woven fabric were characterized using the four-probe method. This technique minimizes the effects of contact and lead resistances, enabling accurate measurement of sheet resistance. With an input current of 10 mA, the voltage drops across the inner voltage electrodes were 60.93 mV, corresponding to a resistance of 6.09 Ω. From this measurement, the sheet resistance was calculated as 27.6 Ω/sq. This reduced sheet resistance confirms the formation of a uniform and conductive PPy coating across the fabric surface.

These values are consistent with those reported for PPy films synthesized via *in situ* chemical polymerization on textile substrates, where sheet resistances typically range from 20–100 Ω/sq depending on the degree of doping and coating thickness. These results validate that the PPy coating developed in this work is suitable for flexible piezoresistive applications, where uniform sheet resistance is essential for consistent electrical signal output under mechanical strain. The linear correlation between applied current and measured voltage demonstrates the ohmic behaviour of the PPy layer, confirming the high quality and uniformity of the conductive coating.

### 3.8 Weight vs. voltage and weight vs. resistance

**FIGURE 7:**
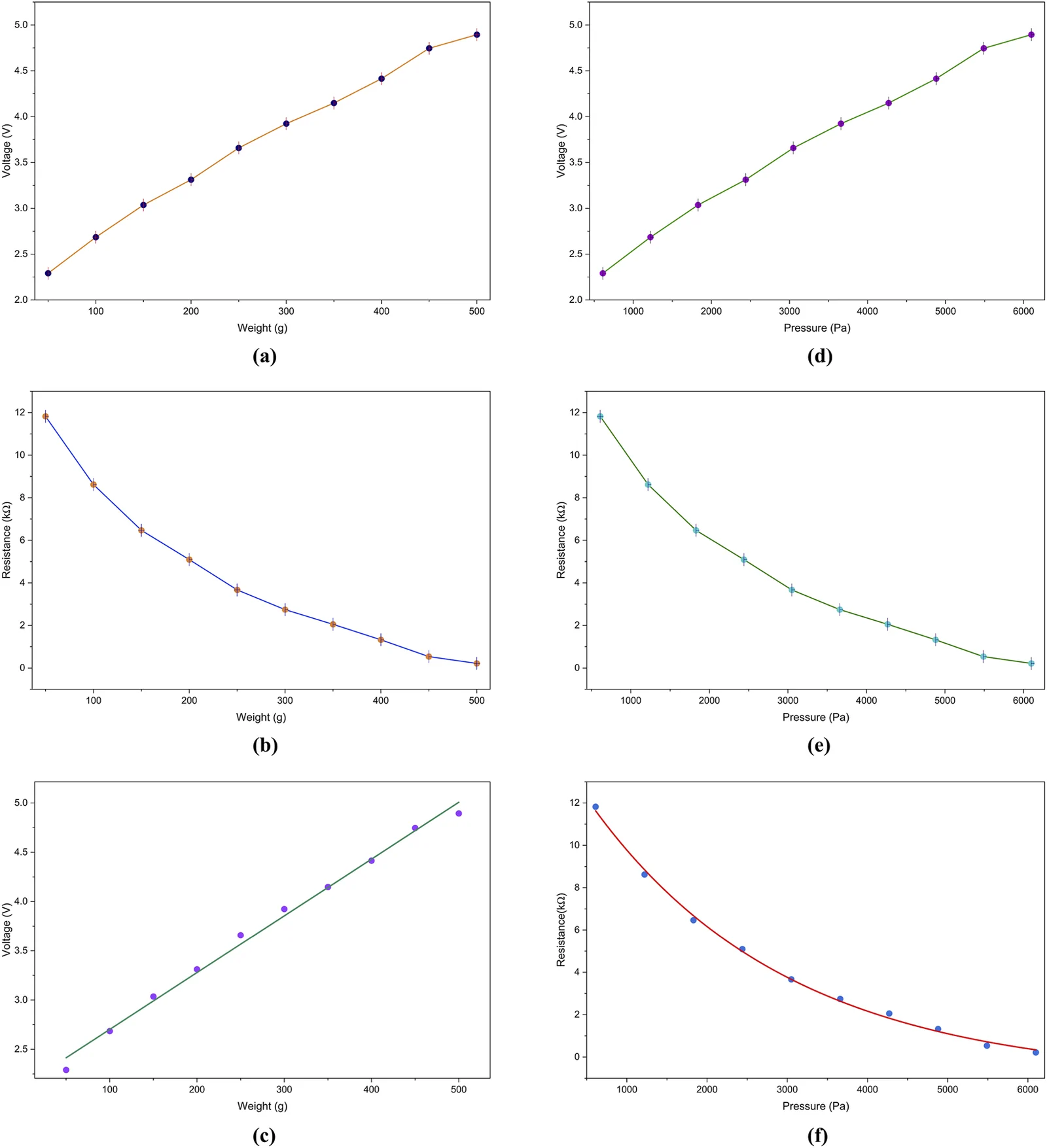
Piezoresistive characterization of PPy-coated nonwoven fabric sensor **(a)** Voltage vs. applied weight; **(b)** Resistance vs. applied weight; **(c)** Linear regression fit of voltage vs. weight, V = 2.127 + 0.00576×W (*R* ^2^ = 0.993, Adj. *R* ^2^ = 0.993, Pearson’s r = 0.997; ANOVA F (1,8) = 1199.7, p < 0.0001); **(d)** Voltage vs. applied pressure; **(e)** Resistance vs. applied pressure; **(f)** Exponential decay fit of resistance vs. pressure, R = −1.01 + 16.21·e^−^ᴾ/^2452^.^9^ (*R* ^2^ = 0.998, Adj. *R* ^2^ = 0.997; ANOVA F (2,7) = 1676.8, p < 0.0001).

The piezoresistive behaviour of the PPy-coated non-woven fabric sensor was investigated by measuring the electrical response under applied weight loading. Figure 7a presents the output voltage as a function of applied weight. The voltage increases progressively from 2.29 V at 50 g to 4.89 V at 500 g, demonstrating a consistent electrical response to mechanical loading with a nearly linear trend across the tested range. Figure 7b shows the corresponding change in electrical resistance with applied weight. An inverse relationship is observed, where resistance decreases from 11.82 kΩ at 50 g to 0.22 kΩ at 500 g. The resistance exhibits a nonlinear decay pattern, with a steeper decline at lower weights that gradually diminishes at higher loads. This behaviour arises from the compression of the PPy-coated fabric structure under applied weight, which enhances inter-fibre contact and creates additional conductive pathways through the PPy network. As load increases, the fabric’s porous structure compresses, bringing more PPy-coated fibres into electrical contact and thereby reducing overall resistance. The complementary trends of increasing voltage and decreasing resistance with applied weight confirm the piezoresistive functionality of the PPy-coated fabric, demonstrating that the sensor transduces mechanical pressure into measurable electrical signals suitable for pressure-sensing applications in wearable and smart textile systems. The carbon-impregnated interlayer additionally functioned as a supportive conductive medium, facilitating charge transport between the top and bottom PPy-coated fabric electrodes during compression.

### 3.9 Pressure vs. voltage and pressure vs. resistance

The piezoresistive response of the PPy-coated fabric sensor was evaluated by measuring electrical output as a function of applied pressure. Applied pressure was calculated from the applied weight using a fixed contact area throughout testing, so the pressure values in this study scale directly with the applied weight (609.885 Pa per 50 g increment) rather than being independently varied. Figures 7a–f therefore present the same underlying measurements, expressed as weight (the directly controlled test parameter) and as pressure (which allows comparison with other piezoresistive sensors in the literature). Figure 7d shows the relationship between applied pressure and output voltage, which increases nearly linearly from 2.29 V at 610 Pa to 4.89 V at 6.1 kPa. Figure 7e presents the corresponding resistance response, which decreases nonlinearly from 11.82 kΩ at 610 Pa to 0.22 kΩ at 6.1 kPa over the same range, consistent with the compression-driven mechanism described in Section 3.8.

### 3.10 Cyclic loading and durability

#### 3.10.1 Repeatability and long-term stability

**FIGURE 8:**
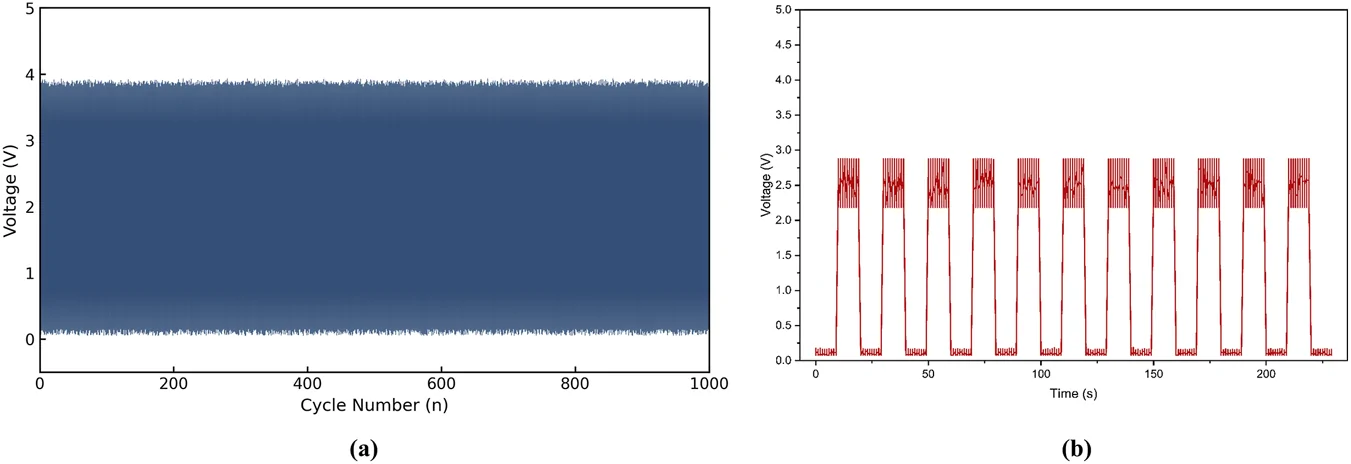
**(a)** Output voltage response recorded continuously over 1000 consecutive loading (300 g) and unloading cycles, showing stable and reversible switching between the loaded (∼3.87 V) and unloaded (∼0.10 V) states with no visible drift throughout the test. **(b)** High-speed monitoring (1000 Hz) of 11 loading-unloading cycles with 100 g weight showing response time <1 ms and recovery time ∼1 s.

Cyclic durability was assessed by subjecting the sensor to 1000 consecutive loading (300 g) and unloading cycles. As shown in Figure 8a, the peak loaded voltage remained stable at 3.867 ± 0.026 V, while the unloaded baseline remained low and consistent at 0.098 ± 0.028 V, giving an average on/off voltage ratio of approximately 39.5. The loaded voltage showed no visible upward or downward trend across all 1000 cycles, indicating the absence of systematic drift or degradation in sensor response over extended cyclic loading. This stability indicates that the conductive PPy coating maintains strong adhesion to the fabric substrate under repeated mechanical deformation.

#### 3.10.2 Dynamic response and recovery characteristics

Dynamic response characterization was performed using high-speed monitoring at a 1000 Hz sampling rate during rapid loading-unloading cycles with a 100 g applied weight. Figure 8b shows 11 complete cycles with consistent transitions between unloaded (0.101 V) and loaded (∼2.50 V) states, representing a voltage change of 2.40 V. The response time (10%–90% rise) was less than 1 ms, indicating instantaneous resistance change upon pressure application due to rapid formation of conductive pathways through fibre-to-fibre contact. The recovery time (90%–10% fall) was ∼1 s, characteristic of viscoelastic textile materials requiring a finite time to return to the uncompressed state after load removal. This asymmetry between response and recovery times reflects the mechanical properties of the textile substrate rather than limitations in electrical conductivity.

### 3.11 Temperature vs. resistance analysis

**FIGURE 9:**
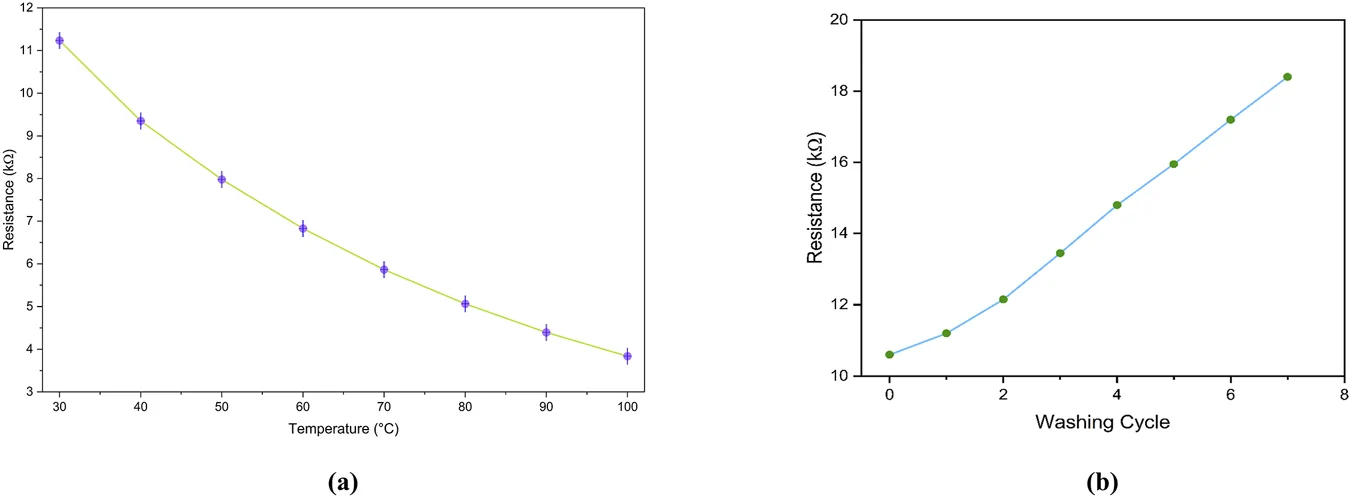
**(a)** Temperature-dependent resistance of the PPy-coated fabric. Resistance decreases from 11.236 kΩ at 30 °C to 3.836 kΩ at 100 °C, fitted with a single-exponential decay model (*R* ^2^ = 0.9996), exhibiting negative temperature coefficient behaviour (TCR = −0.941%/°C). **(b)** Washing durability assessment of the PPy-coated fabric. Resistance increases linearly from 10.6 kΩ (unwashed) to 18.4 kΩ (7 cycles) following AATCC 135 standard, with structural changes becoming evident after 6–7 cycles.

The temperature-dependent electrical characteristics of the PPy-coated fabric were investigated without applied mechanical load. As shown in Figure 9a, the resistance exhibited an inverse relationship with temperature, decreasing from 11.236 kΩ at 30 °C to 3.836 kΩ at 100 °C, representing a 65.9% reduction over the 70 °C temperature range, corresponding to a negative temperature coefficient (NTC) of −0.941%/°C. The temperature-resistance relationship was best described by a single-exponential decay model, R = 1.52 + 17.96·e^−^ᵀ/^48^·^80^ (*R* ^2^ = 0.9996, Adj. *R* ^2^ = 0.9994; ANOVA F (2,5) = 6264.2, p < 0.0001). This NTC behaviour is attributed to thermally activated hopping conduction in PPy, where elevated temperatures facilitate charge transport between polymer chains by overcoming energy barriers, resulting in decreased electrical resistance.

### 3.12 Washing cycle vs. resistance analysis

The washing durability of the PPy-coated fabric was evaluated following AATCC 135 standard protocols with room temperature washing (10 min per cycle with detergent) and air drying. Resistance measurements were recorded after each washing cycle to assess the retention of electrical conductivity. As shown in Figure 9b, the resistance increased progressively from 10.6 kΩ before washing to 18.4 kΩ after seven cycles, representing a 73.6% increase. The resistance evolution followed a highly linear trend (*R* ^2^ = 0.993) with an average increase of approximately 1.1 kΩ per cycle. Through the first five washing cycles, the sensor maintained functional performance with a moderate resistance increase of 50.5% (10.6 kΩ–15.95 kΩ). At the sixth cycle, minor structural changes became visible in the nonwoven PP fabric. By the seventh cycle, more pronounced physical changes were observed, including web thinning, fibre detachment, and surface fuzzing.

The progressive resistance increase is attributed to mechanical stress on the PP nonwoven structure during washing (Aizamddin et al., 2022; Aizamddin et al., 2024), which causes disruption of the conductive PPy network coating. The mechanical agitation induces fibre separation and bond weakening under repeated wetting and drying cycles, reducing the continuity of conductive pathways. Partial delamination of the PPy coating from the fabric surface further contributes to the increased electrical resistance observed with successive washing cycles.

### 3.13 Time vs. voltage analysis

**FIGURE 10:**
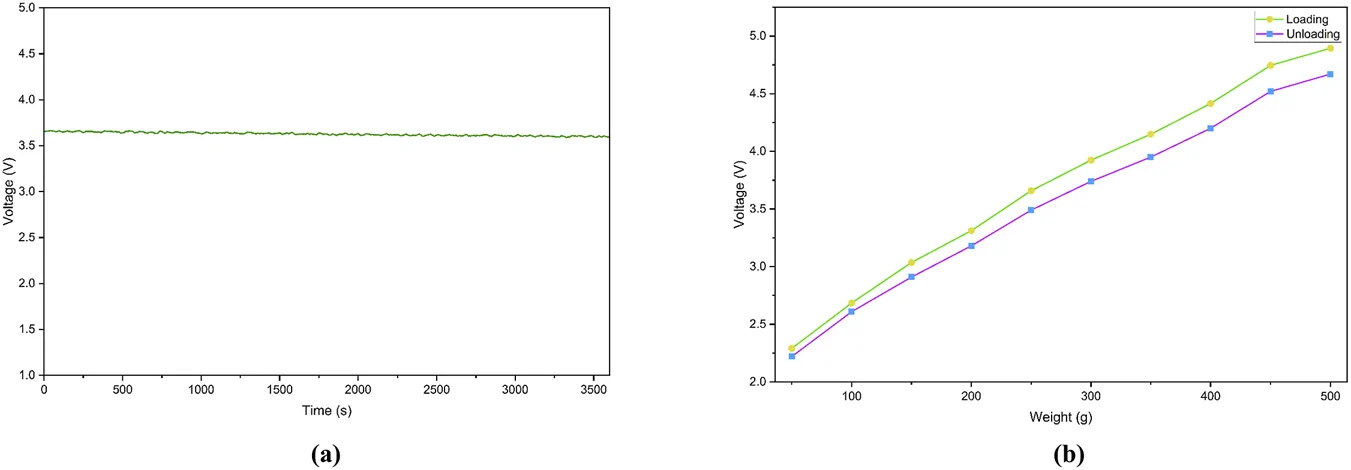
**(a)** Temporal stability of voltage response under sustained loading (250 g) over 1 hour. Mean voltage 3.627 V with coefficient of variation 0.91%, showing a gradual drift of −1.64% attributed to viscoelastic fabric relaxation. **(b)** Hysteresis behaviour during cyclic loading and unloading. Voltage responses show measurable separation between loading (2.291–4.894 V) and unloading (2.220–4.670 V) paths, with maximum hysteresis of 8.68% of full-scale output, typical for textile-based pressure sensors.

The temporal stability of the sensor response was evaluated by continuous voltage monitoring over 1 hour under sustained loading with a 250 g weight. As shown in Figure 10a, the voltage remained relatively stable with a mean value of 3.627 V and a standard deviation of 0.033 V, corresponding to a coefficient of variation of 0.91%. The voltage ranged from 3.522 V to 3.728 V throughout the measurement period. Analysis of the temporal evolution revealed a gradual voltage decrease of approximately 1.64% over the one-hour duration, with the average voltage declining from 3.658 V during the first 100 s to 3.598 V during the final 100 s. This modest drift is attributed to viscoelastic relaxation of the PP nonwoven fabric under continuous compression, which gradually reduces the contact pressure between conductive fibres and decreases the density of conductive pathways. Despite this gradual drift, the coefficient of variation below 1% demonstrates good short-term signal stability suitable for real-time pressure monitoring applications where measurements are acquired over minutes rather than extended hours.

### 3.14 Hysteresis analysis

The hysteresis behaviour of the sensor was evaluated by comparing voltage responses during cyclic loading and unloading under incrementally increasing weights from 50 g to 500 g. As shown in Figure 10b, both loading and unloading curves exhibit a positive correlation between applied weight and voltage output. During loading, the voltage increased from 2.291 V to 4.894 V, while during unloading, the voltage decreased from 4.670 V to 2.220 V, revealing measurable separation between the two curves. The voltage difference between loading and unloading paths represents the hysteresis effect, which is characteristic of viscoelastic materials, where structural relaxation in the polymer-fabric matrix causes a delayed response during pressure release. The hysteresis voltage increased progressively with applied weight, ranging from 0.071 V at 50 g to a maximum of 0.226 V at 450 g, before decreasing slightly to 0.224 V at 500 g. Expressed as a percentage of the full-scale output (2.603 V), the maximum hysteresis corresponds to 8.68%, which falls within the typical range reported for textile-based pressure sensors (5.3%–24%) (Chen et al., 2022; Hao et al., 2020). This moderate hysteresis is characteristic of conductive polymer-coated textile sensors, where the nonwoven fabric substrate undergoes viscoelastic deformation during compression and requires a finite time to recover its original structure upon load removal.

### 3.15 Antibacterial analysis

**FIGURE 11:**
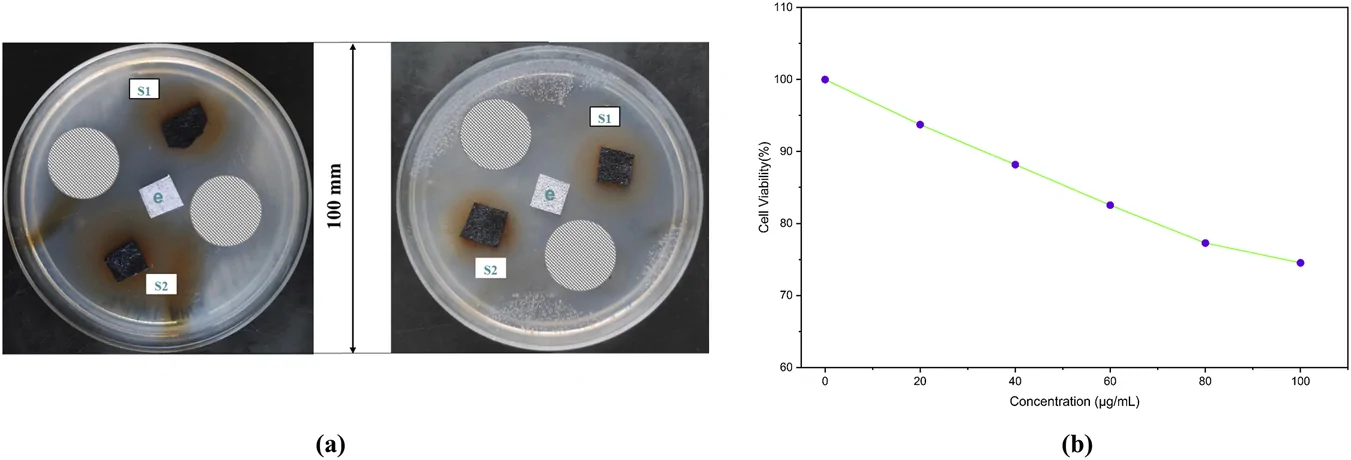
**(a)** Antibacterial activity of PPy-coated fabric against *E. coli* (left) and *S. aureus* (right) using the AATCC 147 method. PPy-coated samples (S1, S2, black) measuring 1 cm × 1 cm show clear inhibition zones (37–42 mm), while the uncoated control (e, white) shows no activity. Both identical replicate samples confirm reproducible antimicrobial effectiveness against Gram-negative and Gram-positive bacteria. **(b)** Cytotoxicity assessment of PPy-coated fabric extract using L929 fibroblast cells. Cell viability decreases in a concentration-dependent manner from 100% (control) to 74.54% ± 1.31% (100 μg/mL). All tested concentrations maintain viability above the 70% threshold defined by ISO 10993–5, confirming non-cytotoxic classification of the material. Data represent mean ± standard deviation from triplicate measurements (n = 3).

As shown in Figure 11a, the PPy-coated samples exhibited clear zones of inhibition measuring 37 mm (S1) and 40 mm (S2) against *E. coli*, and 38 mm (S1) and 42 mm (S2) against *S. aureus*. The uncoated control showed no detectable inhibition, confirming that the antimicrobial activity originates from the PPy coating. The slight variation between replicates was minimal, demonstrating reproducible antibacterial performance. Sample S2 showed marginally higher activity against both bacterial strains, though both samples exhibited comparable effectiveness.

The antibacterial mechanism of PPy arises from electrostatic interactions between the positively charged polymer backbone and negatively charged bacterial cell membranes (Silva Júnior et al., 2020; Da Silva et al., 2016). This binding disrupts membrane integrity, causing leakage of intracellular components and ultimately cell death. The slightly enhanced activity against *S. aureus* may be attributed to differences in cell wall architecture, as Gram-positive bacteria lack the protective outer membrane present in Gram-negative species (Maruthapandi et al., 2022). The demonstrated antibacterial activity represents a beneficial secondary property of the PPy coating beyond its primary pressure-sensing function. In the sensor architecture, the PPy-coated nonwoven fabric layers form the outer surfaces of the assembly (top and bottom layers) with an intermediate conductive layer positioned between them. Both PPy-coated layers are therefore exposed surfaces in the sensor structure. This dual functionality is achieved through the intrinsic properties of the PPy material itself, without requiring additional antimicrobial agents or surface treatments.

### 3.16 Cytotoxicity analysis

The PPy-coated fabric extract exhibited concentration-dependent effects on cell viability (Figure 11b). The control (0 μg/mL) showed 100.00% ± 0.00% viability. At 20 μg/mL, viability remained high at 93.75% ± 1.05%, decreasing progressively to 88.16% ± 1.09% (40 μg/mL), 82.54% ± 0.37% (60 μg/mL), 77.29% ± 0.62% (80 μg/mL), and 74.54% ± 1.31% (100 μg/mL). All tested concentrations maintained cell viability above 70%, the threshold established by ISO 10993–5 for classifying materials as non-cytotoxic and suitable for applications involving biological contact (Mravcová et al., 2017; Ancona et al., 2021). The gradual, dose-dependent decrease in viability indicates that the PPy coating does not induce acute cytotoxic effects within the tested concentration range, confirming the biocompatibility of the PPy-coated fabric.

To position the PPy-coated polypropylene nonwoven pressure sensor within the existing literature, Table 2 presents a comparative analysis with recent textile-based piezoresistive sensors. The comparison covers substrate material, conductive coating, fabrication method, sensitivity, pressure range, durability testing, washing cycles, hysteresis quantification, and biocompatibility validation.

**TABLE 2:** Performance comparison with textile-based piezoresistive pressure sensors.

References	Substrate	Conductive material	Fabrication	Sensitivity	Pressure range (kPa)	Cycles tested	Washing cycles	Hysteresis (%)	Biocompatibility
This study	PP Nonwoven	Polypyrrole	*In-situ* Polymerization	0.47 V/kPa (*R* ^2^ = 0.993) −0.46 kPa^-1^ (*R* ^2^ = 0.916)	0.6–6.1	1000	7	8.68 (FSO)	AATCC 147 ISO 10993–5
(Camlibel and Kandola, 2025)	Cotton and Polyester	Ag/PPy/CNT	Dip Coating	2.31–116 kPa^-1^	0–225	1000	N/A	N/A	N/A
(Lu et al., 2019)	Polyester Nonwoven	GO-PNWF	Dip Coating	23.41 kPa^-1^	0–40	1200	N/A	Low Hysteresis	N/A
(Mahmud et al., 2025)	Electro-Spun Nonwoven	Graphene	Drop Casting	10.5 kPa^−1^	0–50	100	Multiple	Higher Hysteresis	N/A
(Chen et al., 2019)	Polyester/Spandex	Polypyrrole	Interfacial Polymerization	0%–71% strain	N/A	200	N/A	N/A	N/A
(Zhou et al., 2018)	Cotton	AgNW	Dip Coating	2.46 × 10^4^ kPa^−1^ to 5.65 × 10^5^ kPa^−1^	0–30	>1000	N/A	Eeff at 5 kPa was 85.82 kPa	N/A
(Li Y. et al., 2024)	Rayon Nonwoven	MXene/PEDOT:PSS	Screen Printing	754.5 kPa^-1^	0–5.2	1000	N/A	N/A	ISO 10993.6
(Aizamddin et al., 2022)	Polyester	Polyaniline	Immersion method	N/A	N/A	N/A	N/A	N/A	Kirby-Bauer Disk Diffusion

N/A- Not Available.

The present work demonstrates voltage sensitivity of 0.47 V/kPa (*R* ^2^ = 0.993, linear fit) across a pressure range of 0.6–6.1 kPa. The raw resistance-pressure relationship is accurately described by a single-exponential decay model (*R* ^2^ = 0.998; Section 3.9). For comparison with sensitivity values reported in the literature (Table 2), a normalized piezoresistive coefficient of −0.46 kPa^-1^ (*R* ^2^ = 0.916) was additionally derived from a linear fit of the normalized resistance change, [(R_0_−R)/R_0_], against pressure. This normalized coefficient is considerably lower than that of sensors employing specialized nanostructures such as MXene/PEDOT:PSS (754.5 kPa^-1^) or graphene oxide (23.41 kPa^-1^); however, the present work achieves its performance through a simple, single-step *in situ* polymerization process, without the complex multi-step processing or specialized equipment those approaches require. Beyond sensitivity, a key strength of this work lies in the breadth of characterization performed, spanning durability, washing resistance, hysteresis, and biological validation together, whereas the literature typically reports only a subset of these parameters.

This work systematically evaluates 1000 loading-unloading cycles, 7 washing cycles with performance tracking (while most studies report no washing tests), quantitative hysteresis analysis using full-scale output methodology (8.68% FSO, while most studies either report qualitative descriptions such as “low hysteresis” or “higher hysteresis” or do not measure it at all), and biocompatibility validation through both ISO 10993–5 cytotoxicity and AATCC 147 antibacterial testing (while most studies provide no biocompatibility data, with only one study reporting partial ISO testing). The pressure range of 0.6–6.1 kPa covers physiologically relevant pressures reported in the literature for applications such as human touch, finger motion, and joint bending, indicating suitability for wearable motion-monitoring contexts. The trade-off between lower normalized sensitivity and comprehensive validation represents a deliberate design choice toward practical applicability, prioritizing reproducibility, stability, washing durability, and validated biocompatibility over maximum sensitivity optimization that often requires complex hierarchical structures incompatible with scalable manufacturing.

## 4 Conclusion

This work demonstrates polypropylene nonwoven fabric pressure sensors with PPy coating through simple *in situ* polymerization. The sensors respond to pressure across 0.6–6.1 kPa with voltage sensitivity of 0.47 V/kPa (*R* ^2^ = 0.993) and a resistance response accurately described by a single-exponential decay model (*R* ^2^ = 0.998). A normalized piezoresistive coefficient of −0.46 kPa^-1^ (*R* ^2^ = 0.916) is additionally reported for comparison with literature-reported sensors. Performance testing confirms 1000-cycle mechanical durability, one-hour stability with voltage drift of −1.64%, temperature response across 30 °C–100 °C, and retention of function over 7 washing cycles. Hysteresis is 8.68% of full-scale output. Biocompatibility testing shows cell viability maintained above 70% at all concentrations up to 100 μg/mL (ISO 10993–5), and antibacterial activity with inhibition zones of 37–40 mm against *E. coli* and 38–42 mm against *S. aureus* (AATCC 147).

The washing durability was evaluated over 7 cycles, after which the polypropylene nonwoven substrate undergoes physical changes, including fibre splitting and surface morphology alterations. This makes the current sensor suitable for disposable or limited-use applications. Substrate modification or alternative materials could extend washing durability based on specific application requirements.

This work provides a practical approach through simple one-step fabrication, biocompatibility, and systematic characterization across multiple parameters. Future studies should validate performance for applications such as human touch, finger motion, and joint bending in real-world wearable settings. Additional work includes substrate modifications to enhance washing durability and integration into wearable garment systems.

## Data Availability

The original contributions presented in the study are included in the article/supplementary material, further inquiries can be directed to the corresponding author.
